# Draft Genome Sequence of the Versatile Alkane-Degrading Bacterium *Aquabacterium* sp. Strain NJ1

**DOI:** 10.1128/genomeA.01271-14

**Published:** 2014-12-04

**Authors:** Hisako Masuda, Yuh Shiwa, Hirofumi Yoshikawa, Gerben J. Zylstra

**Affiliations:** a School of Sciences, Indiana University Kokomo, Kokomo, Indiana, USA; b Department of Bioscience, Tokyo University of Agriculture, Setagaya-ku, Tokyo, Japan; c Department of Biochemistry and Microbiology, Rutgers University, New Brunswick, New Jersey, USA

## Abstract

The draft genome sequence of a soil bacterium, *Aquabacterium* sp. strain NJ1, capable of utilizing both liquid and solid alkanes, was deciphered. This is the first report of an *Aquabacterium* genome sequence.

## GENOME ANNOUNCEMENT

A substantial amount of *n*-alkanes, which are major components of fossil fuels, have been released in the environment as a result of accidental oil spills and the improper disposal of industrial waste, posing heath concern to humans as well as to wild animals. A number of bacterial strains, capable of metabolizing *n-*alkanes, have been isolated from soil (1). Microbial biodegradation of short- to medium-chain *n*-alkanes is initiated by hydroxylation of the terminal carbon by hydroxylases such as alkane monooxygenase (AlkB) and cytochrome P450 (2, 3). The hydroxylation of longer *n*-alkanes can also be achieved by an additional class of hydroxylase AlmA, as shown in *Acinetobacter* sp. strain DSM 17874 (4). *Aquabacterium* sp. strain NJ1 was isolated from heavily contaminated soil near a gas station in New Jersey, USA. It grows rapidly on liquid and solid linear alkanes as the sole source of carbon. A growing number of culture-independent studies have demonstrated that *Aquabacterium* strains are the predominant members of the microbial community in different places, including drinking water systems, aquifers, human blood, dust storms, and biofilms on various surfaces (5–9). Notably, *Aquabacterium* was identified as a dominant member in hydrocarbon-contaminated soils (10). Despite its prevalence in nature, no genome sequences have been determined for *Aquabacterium* strains.

Whole-genome sequencing was performed using the Illumina platform. Genomic DNA was extracted using the GenElute bacterial genomic DNA kit (Sigma-Aldrich). Sequencing was carried out on an Illumina GAIIx sequencer, generating 4,458,428 reads. *De novo* assembly using CLC Genomics Workbench 6 yielded 327 contigs with an *N*_50_ of 158,098 bp, comprising 5,006,502 bp. With additional PCR and Sanger sequencing, the number of contigs was reduced to 17. The G+C content of the genome was 63.3%. The assembled sequence was annotated by use of the NCBI Prokaryotic Annotation Pipeline.

The genome consisted of 4,362 genes with 4,303 open reading frames, 52 tRNAs, 6 rRNAs, and 1 ncRNA. The genome contained two copies of cytochrome P450-type, five copies of AlkB-type, and eight copies of AlmA-type oxygenases. All five AlkB-type oxygenases contained 8 conserved histidine residues and shared 33% to 53% identity with the protein sequence of well-studied AlkB from *Pseudomonas putida* GPo1 (11). Further research will reveal the physiological roles of each gene in the degradation of *n-*alkanes and other pollutants. Using the RAST SEED viewer (12), the genome was found to share the highest overall similarity with *Methylibium petroleiphiulm* PM1 (13). The gene organizations within contigs, however, differ significantly between these strains, suggesting the occurrence of extensive genome rearrangement events. Numerous transposable elements and conjugative transfer proteins found on strain NJ1’s genome most likely contributed to the observed rearrangements.

### Nucleotide sequence accession number.

The complete genome sequence of *Aquabacterium* sp. strain NJ1 has been deposited in GenBank under the accession no. JRKM00000000.
